# Systematic Review and Meta-Analysis of the Relationship between Actual Exercise Intensity and Rating of Perceived Exertion in the Overweight and Obese Population

**DOI:** 10.3390/ijerph182412912

**Published:** 2021-12-07

**Authors:** Hongli Yu, Chen Sun, Bo Sun, Xiaohui Chen, Zhijun Tan

**Affiliations:** 1 Department of Health and Natural Sciences, Gdansk University of Physical Education and Sport, 80-336 Gdańsk, Poland; bo.sun@awf.gda.pl (B.S.); xiaohui.chen@awf.gda.pl (X.C.); 2 College of Physical Education, Yibin University, Yibin 644000, China; sunchen@yibinu.edu.cn; 3 College of Physical Education, Chengdu Sports University, Chengdu 610041, China; tanzhijun2021@gmail.com

**Keywords:** health-related quality of life, physiological needs, controlling behavior, exercise intensity, physical literacy

## Abstract

The number of overweight (OW) and obese (OB) children, adolescents, and adults has increased globally. Exercise intensity, both actual and perceived, is a significant factor in a variety of health-related investigations and rehabilitation trainings. Despite this, literature on the connection between actual exercise intensity and the rating of perceived exertion (RPE) in overweight and obese populations is lacking. A systematic review, meta-analysis, combined analysis of variance (Brown–Forsythe ANOVA), and Spearman correlation were performed to fill this gap. After preliminary assessments, ten studies were classified as having a low risk of bias and a degree of heterogeneity (I^2^ = 34%; *p* = 0.05). The RPE scores (F = 0.032; *p* = 0.859), physiological index (percentage of maximal heart rate (%HR_max_) (F = 0.028; *p* = 0.869), and percentage of maximal oxygen uptake (%VO_2max_) (F = 2.434; *p* = 0.136) demonstrated consistency without being significantly different between the normal weight (NW) and OW/OB groups. The RPE scores varied by age (NW (coefficient values) = 0.677 ***, OW = 0.585 **), as well as by indoor temperature (OW only, coefficient values = 0.422 *), body mass index (NW (coefficient values) = 0.516 **, OW = 0.580 **), and test time (NW only, coefficient values = 0.451 *). We conclude that RPE is appropriate for the following OW and OB people: (1) those who are older than 21.5 (the lowest age in the group of ≥18) years old and younger than 58.6 (the highest age in the group of ≥18) years old, without any other diseases, and (2) those who engage in low-intensity exercise while maintaining a standard indoor temperature. Future studies may address alternative techniques for increasing the reliability of longitudinal comparisons and gender comparisons, as well as investigate other possible confounding factors.

## 1. Introduction

There are many consequences of being overweight or obese, including health, social, and economic impacts [1,2]. The World Health Organization estimated that obese children and adolescents rose almost tenfold from 11 million in 1975 to 124 million in 2016 [3]. In virtually every area and nation, there was an increasing number of overweight and obese adults reported in 2016, of whom 650 million (13% of the global population) were obese and 1.3 billion were overweight [4]. Obesity may increase the risk of complications in individuals with diabetes, cardiovascular (primarily cardiovascular and stroke), joint pain (particularly degenerative joint arthritis), and some malignancies (endometrial cancer, breast cancer, etc.) [4,5]. Indeed, these obesity-related problems are particularly prevalent in children, teenagers, and adults [6].

Multiple studies have found that moderate- to high-intensity physical exercise is critical for several additional therapeutic benefits [7,8], including improved health-related fitness [9,10], a healthy weight status [9,11], and psychological well-being [12,13,14]. However, being overweight (OW) and obese (OB) typically entail unmatched energy consumption and expenditure that are generally linked with insufficient physical exercise intensity [15,16,17]. Intensity refers to the force exerted by a movement and the amount of physical tension present, and it is one of the primary drivers of exercise load [18,19]. Adequate intensity may considerably enhance the body’s functions and may increase fitness [20,21,22]. When intensity exceeds the body’s capacity to endure, the body’s ability to operate declines and even health is endangered [21,23]. Therefore, OW and OB individuals must engage in the rapid and efficient monitoring of exercise intensity in the process of investigating the relationship between exercise intensity and obesity, because rapidly and efficiently measuring exercise intensity helps people with obesity to timely evaluate exercise intensity and scientifically participate in exercise [24,25]. Exercise intensity is also a critical component in developing reasonable and efficient exercise prescriptions for these individuals [25]. People may decrease obesity rates in a healthy and effective manner with the assistance of a reasonable and effective exercise prescription.

Exercise intensity plays a vital role in the factors mentioned above. It has subsequently led to an increase in relevant studies of exercise intensity [18]. Traditionally, exercise intensity in studies has been determined using physiological indicators (e.g., heart rate (HR), oxygen uptake (VO_2_), maximum heart rate (HR_max_), and maximal oxygen uptake (VO_2max_)) [26,27]. However, these results cannot be verified unless expensive equipment and professional supervision are used, because they are difficult to assess [28]. One correlation of people’s exercise intensity is the Borg’s rating of perceived exertion (RPE) scale (e.g., Borg, 1973, 1982, 1998). RPE represents proprioception exercise intensity, i.e., a numerical estimation of exercise intensity. The score was originally based on the Borg scale [29,30,31] and has been shown to be an effective tool for this prediction [32,33]. The Borg scale has been extensively utilized in medicine for over 40 years and is still in widespread usage today [29]. Exercise physiologists and physicians utilize the scale to interact with patients during exercise testing, and participants may instantly express their level of subjective effort [29]. Fitness coaches may also utilize this method while teaching students, either alone or in combination with a heart rate monitor, to determine the proper intensity of exercise [29]. In the context of this review, the term RPE is used to refer to an individual perception (i.e., personal identification and interpretation of effort exerted) of their actual exercise intensity (AEI) level [31].

Due to the increased attention to RPE and exercise intensity, numerous studies have been conducted to determine the relationship between an individual’s AEI and RPE, because it has been verified that RPE could have a more accurate assessment of the real intensity of exercise in health individuals, thus increasing the validity and reliability of studies [34,35,36]. For instance, the *Journal of Motor Learning and Development* recently ran a special edition consisting of five studies that attempted to answer whether AEI correlates with RPE [37,38,39,40,41]. Some, but not all, investigations into the association between normal weight (NW), OW, and OB revealed significant positive associations between AEI and RPE [42,43,44,45]. It is also difficult to conclude the degree of connection between OW and OB people’s AEI and RPE due to the disparate findings and the absence of an overview of the relationship between AEI and RPE in OW and OB groups. Consequently, it is difficult to say if the Borg scale is valid and reliable across various groups of people.

In addition, when forming conclusions based on research findings, it is important to consider variables (e.g., intrapersonal characteristics, operationalization of AEI, and the degree of alignment). For instance, the strength of the connection between AEI and RPE may be influenced by individuals’ intrapersonal variables, because RPE is a developing phenomenon that improves through developmental time in lockstep with overall cognitive growth [36]. Second, prior research has indicated that OW and OB people sweat more than normal weight (NW) people at the same temperature and intensity [46], limiting their capacity to properly assess their AEI [47]. Third, it is anticipated that AEI connections may grow stronger with age and body mass index (BMI) [46]. Fourth, the strength of the connection between AEI and RPE may depend on how AEI (e.g., exercise modalities, surroundings, and protocols) and RPE are operationalized (e.g., test time and measurement of the perceived exertion scale). The operationalization of AEI and RPE determine their degree of alignment, thus affecting the connection between the two constructs [26].

Obesity is now a worldwide concern with health issues that urgently need to be addressed. RPE has been extensively utilized in scientific studies (e.g., utilized in different questionnaires) and rehabilitation training assessment (e.g., exercise prescriptions) [48,49]. However, the reliability and validity of RPE in OW and OB populations is debatable, and no systematic research has been conducted yet. Meanwhile, the limited sample size of a single article and the lack of statistical significance in the differences between groups need to be tackled. Furthermore, RPE’s reliability is restricted by geographical location, time, and the research object in a single study. Therefore, we created different designs for this research based on the information provided above. First of all, OW and OB individuals without other diseases were included as study objects. Secondly, a meta-analysis is compatible with people’s concept of objective rules and fully congruent with the principle of evidence-based medicine. Thus, a meta-analysis combined with a systematic review was conducted for this paper. As such, in comparison to individual studies, our sample size was increased, and our statistical significance of inter-group differences was more apparent. The accuracy of statistical efficacy and effect estimations enabled more objective and accurate evaluations and accounts for the heterogeneity of various findings. Thirdly, though our innovation point is presented above, we also investigated the impact of moderator factors (e.g., age and BMI, intrapersonal characteristics, and operationalization of AEI) on RPE, which offers a reference foundation for future studies of RPE applications in OW and OB populations that will make it possible to draw more explicit, new, and reliable conclusions.

As a result of the abovementioned factors, it remains unknown (a) how OW and OB individuals’ objectively assessed AEI values correlate with their RPE values and (b) whether this connection may diverge according to intrapersonal variables (e.g., age and BMI), RPE measuring, exercise modalities, or protocol for exercise. Thus, the objectives of this review were (1) to conduct a comprehensive examination and synthesis of scientific data on the association between AEI and RPE in OW and OB individuals and (2) to investigate various a priori identified possible mediators of the connection between AEI and RPE (e.g., age, BMI, exercise categories, and exercise protocols). Based on the explanations mentioned above, it was predicted that (1) research that utilizes perception measures that correspond to real exercise intensity assessment have greater correlations, (2) the strength of these relationships grows as individuals become older, (3) these associations may vary with BMI, and (4) associations may be stronger in different exercise types and protocols.

## 2. Materials and Methods

### 2.1. Protocol and Registration

The current systematic review was conducted following the preferred reporting items for systematic review and meta-analysis (PRISMA) statement [50]. It was registered with PROSPERO, the international database of prospectively registered systematic reviews in health and social care, on 29 August 2021 (CRD42021270819). The review protocol can be accessed via https://www.crd.york.ac.uk/prospero/display_record.php?ID=CRD42021270819.

### 2.2. Search Strategy

Between March and May 2021, a systematic literature search for primary studies was conducted on EBSCOhost using the databases of MEDLINE, SPORTDiscus, Academic Search Ultimate, Health Source: Nursing/Academic Edition, and MasterFILE Premier. An advanced search was utilized; the search engine included Boolean/-phrase expanders applied to comparable topics and negative exclusion for papers that were not peer-reviewed or in English language. The subjects were overweight and obese. Selection criteria were (1) no age, gender, or year of publishing limitations (2) and a fit for our chosen topic. “and” and “or” were among the major Boolean operators used. The words “overweight” or “obese” or “obesity” or “fat” or “unhealthy weight” or “high BMI” or “high body mass index” were used in conjunction (and) with the terms “RPE” or “rating of perceived exertion” or “perceived exertion” or “Borg scale” or “OMNI”.

### 2.3. Eligibility Criteria

Starting with the first stage, we removed duplicates and unnecessary titles, and we omitted non-full-text articles. In the second phase, two separate researchers (Y.H.L. and X.C.) scanned abstracts for possibly important information and then used the population, intervention, comparison, outcomes and study design (PICOS) process [51]. The PICOS process is a formatted retrieval method based on evidence-based medicine (EBM) that quickly and effectively excludes irrelevant articles [52]. A third stage was employed to acquire full texts matching abstracts that fulfilled the eligibility requirements and likely relevant articles: full texts corresponding to abstracts that satisfied eligibility criteria and reference list articles were found. The fourth stage was a thorough examination of complete articles for possible inclusion, and the articles were rated for their eligibility. When two investigators could not agree on whether or not to include an item in the research, the senior author took on the additional duty of conducting an independent evaluation to determine whether the article was suitable for inclusion.

The full-text screening was conducted following the criteria below:

1. The study sample included the overweight and/or obese population without other diseases and had no age or sex limitations.
2. The study included an assessment of AEI. There were no restrictions on exercise protocol, type or interval time, or intensity.
3. The study included an assessment of RPE. There were no restrictions on the scale (e.g., Borg scale (0–10), OMNI scale, and Borg scale (6–20)).
4. The study analysis reported on the assessment of the relationship between AEI and RPE.
5. The study had to include randomized controlled trials of NW and OW or OB individuals.
6. To ensure the quality of the included articles, the impact factor of the articles had to be no less than 2.

### 2.4. Data Extraction

Data were extracted from the chosen articles. The extracted data included authors, year of publication, the country in which the study was conducted, study population (i.e., number of subjects in each group, age range (mean and standard deviation (SD)), sex (males and females), BMI (mean and SD)), perceived exertion scores (mean, SD, mode, and interval time (min)), exercise (type, intensity, total time (min), and protocol), temperature, physical signs (e.g., HR (beat.min^-1^ in mean and SD) and VO_2_ (mL/kg/min in mean and SD)), statistical methods, and results.

### 2.5. Methodological Quality and Risk of Bias Assessment

Two independent reviewers used the consensus-based standards for the selection of health measurement instruments (COSMIN) risk of bias tool to test for the presence of bias and the methodological quality of included studies. The COSMIN risk of bias tool provides criteria for determining if a study’s results can be trusted. To evaluate study quality, each standard must be evaluated. The worst-score-count approach must be used to estimate the risk of bias (standards that are not relevant are excluded from the final rating) [53]. The phrase ‘risk of bias’ is a technique for the systemic review of procedures and diagnostic testing precision studies, as per Cochrane [53]. The 10 criteria that comprise the box comprise the fundamentals of patient-reported outcome measure (PROM) development, content validity, structural validity, internal consistency, cross-cultural validity/measurement invariance, reliability, measurement error, criterion validity, hypotheses testing for construct validity, and responsiveness [53]. Using a 4-point scale (i.e., very good, adequate, doubtful, or inadequate), the 10 components were assessed. For certain standards, the answer choice ‘NA’ (not applicable) may be problematic. For instance, if research on structural validity is based on classical test theory (CTT), the criterion for item response theory (IRT) is inapplicable and therefore should not be included in the study “worst score counts” assessment [53]. The cells in this standard have a grey background and should not be utilized [53].

### 2.6. Data Analyses

We performed a meta-analysis and variance analysis (Brown–Forsythe ANOVA) and included a Spearman correlation analysis. The Brown–Forsythe ANOVA was used to determine the differences in RPE scores and physiological indexes (i.e., percent of maximal heart rate and percentage of maximal oxygen uptake) between the NW and OW or OB groups. Spearman correlation analysis was used to study the correlation between intrapersonal factors (such as age and BIM), exercise type, exercise protocol, and RPE scores in all NW and OW or OB group studies. The final mean values for each group were examined together with their standard deviations for each outcome. Weights for the research were determined using the inverse variance technique. Depending on the sample size, the effect size was identified as Hedges’ g. The deviation correction factor was precisely calculated and the effect size using the Hedges and Olkin correcting standard errors.

The Cochran test was used to determine statistical heterogeneity among publications, and inconsistency was assessed using I^2^ statistics, defined as I^2^ = 100% (Q-DF)/Q, where Q is Cochran’s heterogeneity index and DF denotes degrees of freedom [54]. A value of 0% implies that there is no heterogeneity, whereas a larger value shows the existence of heterogeneity [54]. For analyses where I^2^ was less than 50%, a fixed-effects model was used; if I^2^ was more than 50%, a random-effects model was employed [53]. A funnel plot was constructed to evaluate publication bias, and a sensitivity analysis and an Egger test were used. A 95% confidence interval (95% CI) was deemed significant according to the analysis conducted using Stata software version 16.0 (StataCorp, College Station, TX, USA).

## 3. Results

### 3.1. Included Studies

**Figure 1:**
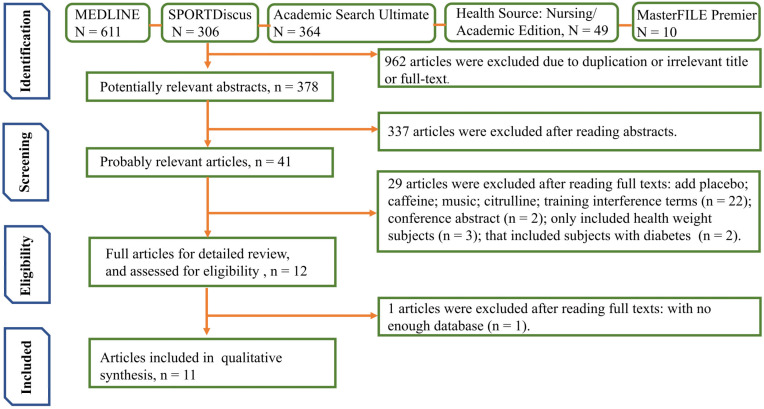
Preferred Reporting Items for Systematic Reviews and Meta-Analyses (PRISMA) flow diagram of the study selection process and results.

A total of 1340 articles were found, 611 of which were from MEDLINE, 306 were from SPORTDiscus, 364 were from Academic Search Ultimate, 49 were from Health Source: Nursing/Academic Edition, and 10 were from MasterFILE Premier. After eliminating duplicates, irrelevant titles, and papers that were not accessible in full form, 378 potentially relevant studies remained. Following the reading of the abstracts, 41 potentially relevant publications were read in full, and 11 studies were selected for review. The search process shown in Figure 1 explains the method of exclusion and the reasons behind it.

Table 1 summarizes the main features of the papers that contributed to the systematic review findings concerning the authors, setting, sample characteristics (i.e., sample size, age and BMI range, sex, exercise type, and protocol), and end measure features (i.e., physiological index and RPE scale).

Each study was a randomized controlled trial (RCT) (n = 741; 100%). The years of publication ranged from 2002 to 2019. These studies included between 21 and 300 individuals; of the 741 subjects in 11 studies, 636 (85.8%) were female, and 105 (14.2%) were male. In this study, participants’ ages ranged from younger than or equal to 18 (n = 219; 29.6%) to over 18 (n = 522; 70.4%), while their weights ranged from normal (n = 226; 30.5%) to overweight (n = 88; 11.9%) to obese (n = 427; 57.6%). Additionally, six papers (n = 542; 73.1%) utilized a continuous progressive exercise regimen [55,57,59,61,62,63], whereas five studies (n = 199; 26.9%) used an intermittent progressive exercise methodology [56,58,60,64,65]. Eight studies (n = 617; 83.3%) used low intensity exercise [56,57,58,60,61,63,64,65], two studies (n = 103; 13.9%) used moderate intensity exercise [59,62], one study (n = 21; 2.8%) used severe intensity exercise [55], and four studies (n = 156; 21.1%) utilized standard temperature exercise [56,57,59,65]. The mode of exercise consisted of the exercise of a 20 min grade treadmill in four articles (n = 217; 29.3%) [56,58,59,61], sub-maximal cycling for 20 min in four articles (n = 386; 52.1%) [57,60,62,63], 45 min school physical activity in one article (n = 52; 7%) [62], running and kicking in one article (n = 25; 3.4%) [65], and the exercise of resistance in one article (n = 61; 8.2%) [64]. Additionally, one article used the Borg scale from 0 to 10 (n = 60; 8.1%) [62], eight articles used the Borg scale from 6 to 20 (n = 595; 80.3%) [55,56,57,58,59,60,61,65], two articles used the OMNI scale (n = 86; 11.6%) [63,64], four articles tested the PE scale at the end of exercise (n = 198; 26.7%) [55,62,64,65], two articles tested each 5 min (n = 46; 6.2%) [56,63], four articles tested at the last 15 s (n = 475; 64.1%) [57,58,59,61], and one article recorded 55 s (n = 22; 3%) [60]. When compared to the utilized reference criteria, five studies considered HR and VO_2_ (n = 457; 61.7%) [56,57,58,61,62], six studies considered VO_2_ (n = 522; 70.4%) [56,57,58,59,60,62], and eight studies considered HR (n = 615; 82.3%) [55,56,57,58,61,62,63,65].

### 3.2. Bias Risk Assessment

**Figure 2:**
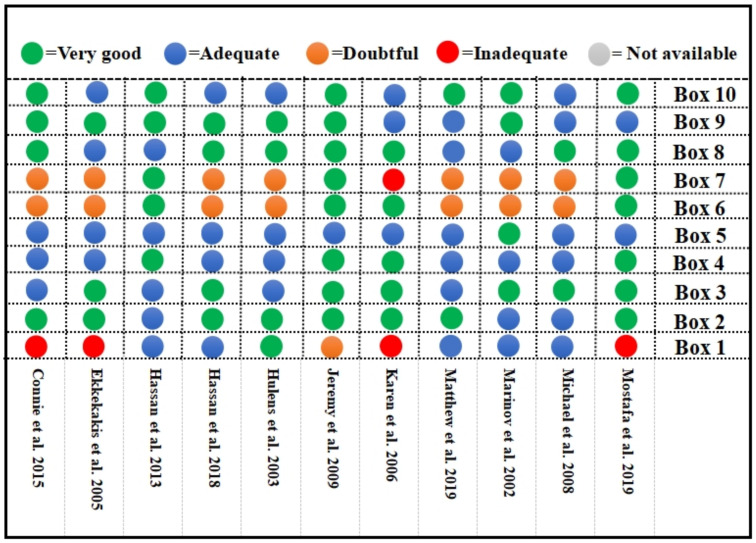
Assessment of study quality and risk of bias. Box 1: patient-reported outcome measure (PROM) development; Box 2: content validity; Box 3: structural validity; Box 4: internal consistency; Box 5: cross-cultural validity\ Measurement invariance; Box 6: reliability, Box 7: measurement error; Box 8: criterion validity; Box 9: hypotheses testing for construct validity; Box 10: responsiveness. The grade (very good, adequate, doubtful, or inadequate) of each box refers to the consensus-based standards for the selection of health measurement instruments (COSMIN) risk of bias tool [53].

The intraclass correlation coefficient (ICC) was calculated to be more than 0.85 among reviewers. Decisions were made based on consensus for dissenting pieces. Ten studies were characterized as having a low risk of bias in all areas, except for that of Hassan et al. [57]. The bias was mostly due to inconsistency in the design requirements for reliability (Box 6) and measurement errors (Box 7), as well as the sample size used in PROM development (Box 1). A single study [60] demonstrated unsatisfactory measurement errors due to SD from another population (%) in its statistical methods. The domain of design requirements demonstrated doubtful minor methodological flaws because only four articles [57,59,60,65] controlled the temperature. In contrast, two studies [57,59] requested a normal diet without alcohol. A significant risk of bias also arose from the sample size employed in four of the included papers; the sample size was adequate in just five (45.5%) of the studies (Figure 2).

### 3.3. Meta-Analysis

#### 3.3.1. Heterogeneity Test and Pooled Results

**Figure 3:**
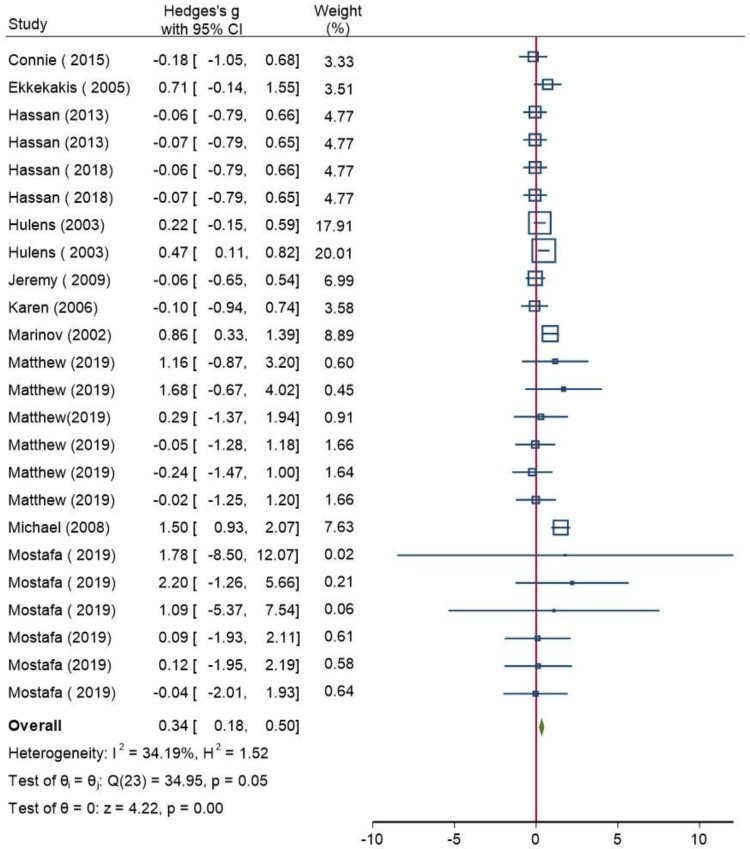
A forest plot of the meta-analysis that compares the rating of perceived exertion scores between the normal weight group and the overweight/obese group. CI: confidence interval, I^2^: the level of heterogeneity, H^2^: the level of heterogeneity, Q: Cochran’s Q heterogeneity test statistic, θ: overall effect size, p: *p*-value for test of significance of overall effect size, Z: z statistic for test of significance of overall effect size. These squares represent the individual studies effect, larger size has more weight. The blue line represents the 95% confidence interval. The diamond represents the overall effect. The red line is line of no effect.

**Figure 4:**
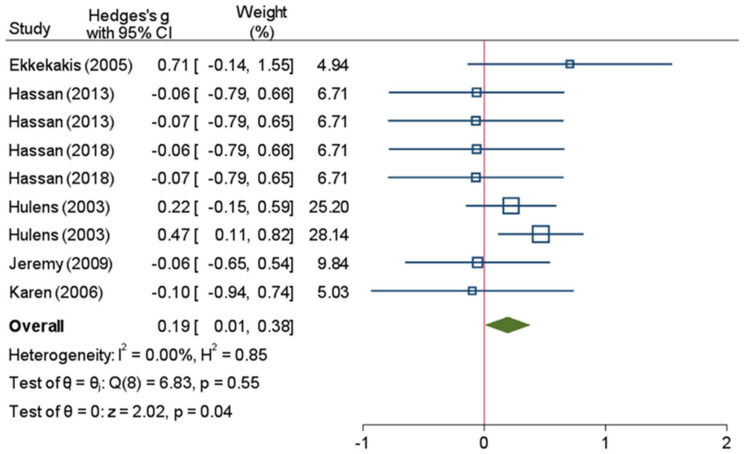
A forest plot of meta-analysis that compares the rating of perceived exertion scores between the normal weight and overweight/obese groups (age ≥ 18). CI: confidence interval, I^2^: the level of heterogeneity, H^2^: the level of heterogeneity, Q: Cochran’s Q heterogeneity test statistic, θ: overall effect size, p: *p*-value for test of significance of overall effect size, Z: z statistic for test of significance of overall effect size. These squares represent the individual studies effect, larger size has more weight. The blue line represents the 95% confidence interval. The diamond represents the overall effect. The red line is line of no effect.

**Figure 5:**
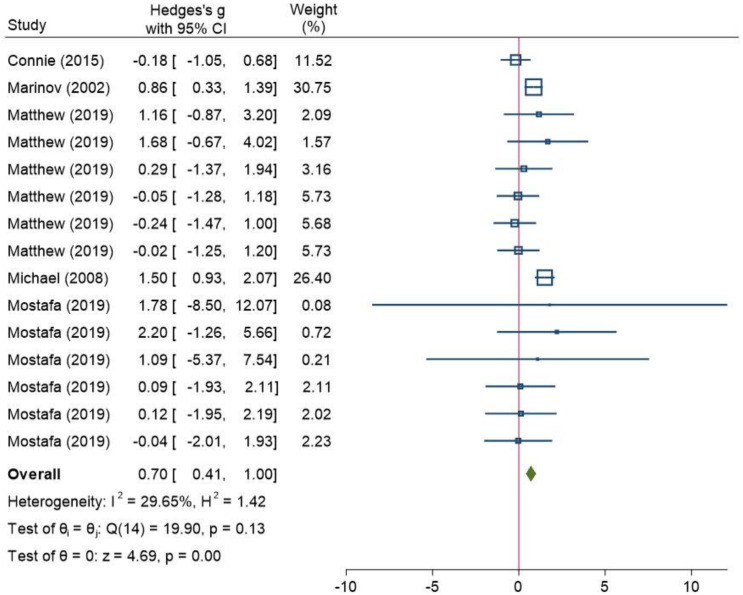
A forest plot of meta-analysis that compares the rating of perceived exertion scores between the normal weight and overweight/obese groups (age < 18). CI: confidence interval, I^2^: the level of heterogeneity, H^2^: the level of heterogeneity, Q: Cochran’s Q heterogeneity test statistic, θ: overall effect size, p: *p*-value for test of significance of overall effect size, Z: z statistic for test of significance of overall effect size. These squares represent the individual studies effect, larger size has more weight. The blue line represents the 95% confidence interval. The diamond represents the overall effect. The red line is line of no effect.

Overall, 11 studies (741 participants) assessed RPE scores (i.e., Borg scale of 6–20, Borg scale of 0–10, or OMNI) (Table 1). Because one study [63] investigated the two case studies and one study [65] designed two-property measurements, both from the same treatment and control group, three studies [57,58,61] used two treatments in the same control group. Consequently, a common model was employed to analyze the data to guarantee the dependability of the findings. The participants in the test group who were overweight or obese had substantially higher RPE values than those in the standard-weight control group (Hedges’s g of 0.34 (95% CI: 0.18, 0.5)). The result was statistically significant, and the heterogeneity was modest, with an I^2^ of 34% and a *p*-value of 0.05 (Figure 3). Subgroup analysis was conducted based on age groups (i.e., ≥18 and <18). The findings revealed no heterogeneity within the age group of ≥18 (I^2^ = 0%; *p* = 0.55) (Figure 4), but a low degree of heterogeneity (I^2^ = 30%; *p* = 0.13) was seen in the age group of <18 (Figure 5).

**Figure 6:**
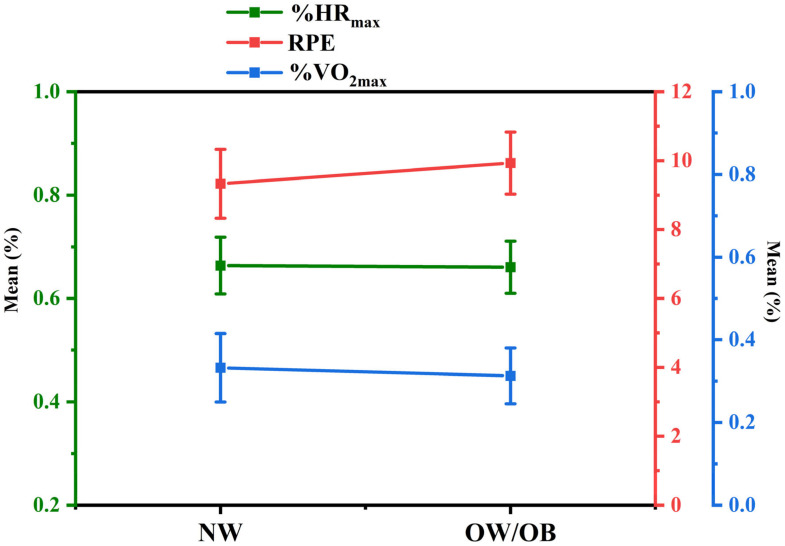
Point chart for comparison of percentage of maximal heart rate, percentage of maximal oxygen uptake, and rating of perceived exertion scores differences between normal and overweight/obese groups. NW: normal weight; OW/OB: overweight/obese; %HR_max_: percentage of maximal heart rate; %VO_2max_: percentage of maximal oxygen uptake; RPE: rating of perceived exertion scores.

In addition, the physiological outcomes of percentage of maximal heart rate (bpm) (%HR_max_) and percentage of maximal oxygen uptake (ml/kg/minute) (%VO_2max_) were used as reference criteria. In three studies [59,60,64], HR was not measured, while in four publications [55,63,64,65], VO_2_ was not measured. Figure 6 illustrates the differences between RPE scores, %HR_max_, and %VO_2max_ as determined by the Brown–Forsythe ANOVA): diverse BMI samples did not significantly vary in %HR_max_ (F = 0.028; *p* = 0.869), RPE scores (F = 0.032; *p* = 0.859), and %VO_2max_ (F = 2.434; *p* = 0.136), suggesting that different BMI samples demonstrated consistency in RPE scores, %HR_max_, and %VO_2max_ (Figure 6).

**Figure 7:**
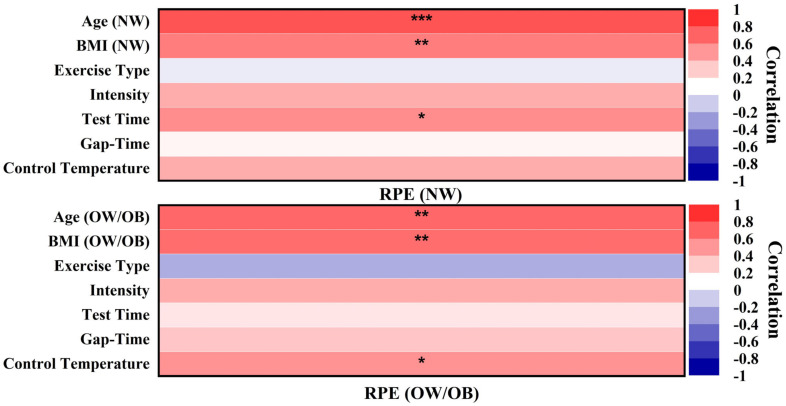
Heat maps of Spearman correlation of rating of perceived exertion scores and age, BMI, exercise type, intensity, test time, gap-time, and control temperature. The normal weight group is shown in the first map. The overweight/obese group is shown in the second map. NW: normal weight; OW: overweight; OB: obese; BMI: body mass index; RPE: rating of perceived exertion scores; ***: significant correlation at *p* < 0.001; **: significant correlation at *p* < 0.01; *: significant correlation at *p* < 0.05. The darker color, the stronger the connection and vice versa; red is positively linked, whereas blue is negatively connected.

Three variables, age (NW), BMI (NW), and test time (test RPE time), were found to be significant, and their correlation coefficients were all higher than 0, indicating a positive connection between age (NW), BMI (NW), test time, and RPE (NW) scores. Exercise type, intensity, gap-time (continuous or interval during exercise), and control temperature (indoor temperature) were shown to have no statistically significant relationship with RPE (NW) scores. The correlation values were close to 0, and all the *p*-values (representing the comparison of exercise intensity, exercise type, gap-time, and control temperature with RPE (NW) scores) were >0.05, suggesting that there was no connection with RPE (NW) scores (Figure 7).

Three variables, age (OW/OB), BMI (OW/OB), and temperature, were found to be significant, and their correlation coefficients were all higher than 0, indicating that there was a positive association between age (OW/OB), BMI (OW/OB), control temperature, and RPE (OW/OB) scores. No significant connection was seen between exercise type, intensity, gap-time, test time, and RPE (OW/OB) scores. Connection coefficients were near 0, and all *p*-values were more than 0.05, suggesting that no correlation existed between exercise type, intensity, gap time, test time, and RPE (OW/OB) scores (Figure 7).

#### 3.3.2. Sensitivity Analysis and Publication Bias

**Figure 8:**
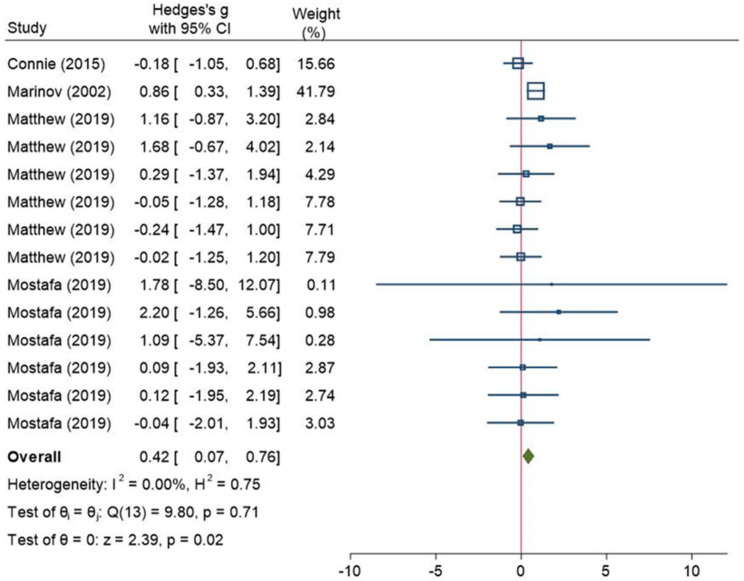
A forest plot of the meta-analysis that compares the rating of perceived exertion scores between the normal weight group and the overweight/obese group (age < 18) after the removal of the study by Michael et al. [64]. CI: confidence interval, I^2^: the level of heterogeneity, H^2^: the level of heterogeneity, Q: Cochran’s Q heterogeneity test statistic, θ: overall effect size, p: *p*-value for test of significance of overall effect size, Z: z statistic for test of significance of overall effect size. These squares represent the individual studies effect, larger size has more weight. The blue line represents the 95% confidence interval. The diamond represents the overall effect. The red line is line of no effect.

**Figure 9:**
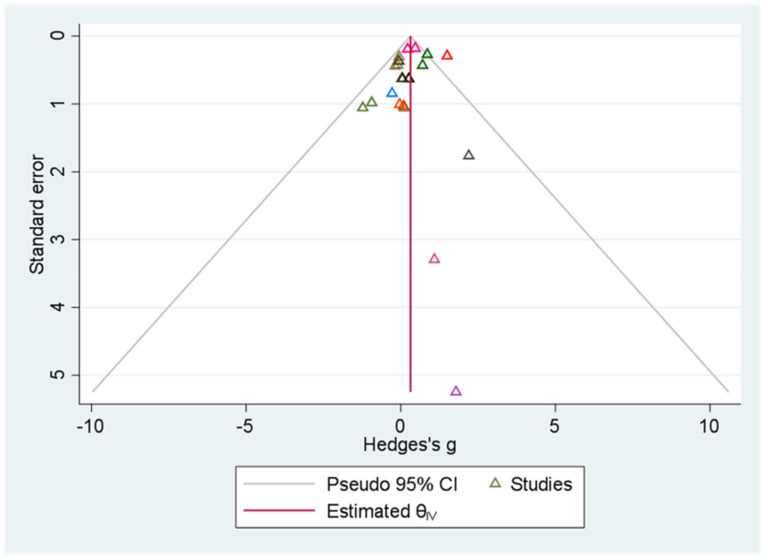
Funnel chart for analyzing the outcome of the rating of perceived exertion scores. Triangles of the same color represent different control design results in the same study.

Sensitivity analysis was performed to ascertain the possibility of outliers and/or influencing studies. Significant differences were discovered when the independent sample *t*-test was employed, necessitating the exclusion of the study by Michael et al. [64], the sole study that posed a risk of bias in measurement error [60], and studies that changed the fixed model or used standard mean difference. All that was altered was the removal of the study by Michael et al. [64], which reduced heterogeneity (Hedges’ g of 0.42 (95% CI: 0.07, 0.79); *p* = 0.02; I^2^ = 0%; *p* = 0.71) (Figure 8). The funnel plot and Egger’s test were used to evaluate the small-study effect and publication bias. Overall, we found no evidence of publication bias in any of the research. According to the funnel plot (Figure 9), there was no indication of asymmetry or publication bias according to the Egger test (*p* = 0.80).

## 4. Discussion

This systematic review and meta-analysis were aimed to characterize the strength of the relationship between AEI and RPE in OW and OB individuals. It is the first review and meta-analysis intended to better comprehend and scrutinize the connection between the two concepts. The authors of this study also investigated the impact of moderator factors (e.g., age and BMI, intrapersonal characteristics, and operationalization of AEI) on RPE. Overall, the primary hypothesis regarding a positive link between AEI and RPE in OW and OB individuals was validated. The RPE scores of OW and OB individuals were found to be greater than normal weight individuals, resulting in an overestimation of exercise intensity. However, there was no significant difference in RPE scores between NW and OW/OB (F = 0.032; *p* = 0.859). The heterogeneity was modest (I^2^ = 34%; *p* = 0.05). Subgroup analysis revealed no heterogeneity within the age group of ≥18 (I^2^ = 0%; *p* = 0.55), but a low degree of heterogeneity (I^2^ = 30%; *p* = 0.13) was seen in the age group of <18. Sensitivity analysis revealed that just one piece of research [64] was influential, since there was no heterogeneity when it was removed from the age group of <18.

With the exception of that of Hassan et al. [57], 10 studies were classified as having a low risk of bias across all domains. In contrast, one study [60] was found to be deficient in terms of measurement error, and the sample size was only acceptable in 45.5% of the studies. Furthermore, the physiological outcomes of %HR_max_ (F = 0.028; *p* = 0.869) and %VO_2max_ (F = 2.434; *p* = 0.136) during exercise revealed no significant differences between the NW group and the OW or OB groups. Indoor temperature, BMI, and age were all linked with RPE scores (OW/OB) and sequentially increased.

### 4.1. Overview of Hypothesis Validation Findings

We predicted that there would be significant correlations between all components of the AEI and RPE in either the OW or OB groups. The review and meta-analysis verified this hypothesis. The RPE scores of OW and OB were found to be greater than those of normal weight individuals, resulting in an overestimation of exercise intensity. However, there was no significant difference in RPE scores between NW and OW/OB. The validity and reliability of RPE in OW and OB people was also fully affirmed. To further verify the conclusion, in this research, ANOVA was used to compare physiological indexes and perceptual ratings between the NW and OW/OB groups throughout the study. As previously stated, using physiological indicators as reference values can allow for the correct assessment of actual exercise intensity during exercise. Based on the available data, no significant variations in either physiological or perceptual outcomes were identified between the two groups, suggesting that the real intensity of motion and perceived values were uniform. However, throughout the systematic review process, we discovered that 10 studies utilized distinct physiological indicators, with HR and VO_2_ being the most often used. As a result, %HR_max_ and %VO_2max_ were used as actual reference indexes in this paper. Still, some studies only measured HR or VO_2_, and a few studies simultaneously measured multiple physiological indexes (e.g., (tidal volume) VE and (minute ventilation) VT). One study did not use any physiological indicators as a reference [64]. Inconsistent use of physiological parameters may influence study findings, particularly when results are longitudinally compared. For example, age may affect HR [66], and BMI may affect VO_2_ [67,68]. Thus, we suggest that the use of physiological indicators in longitudinal comparisons requires caution in future studies.

We anticipated that a variety of variables influenced the RPE scores. We found that indoor temperature, BMI, and age were all linked with RPE scores (OW/OB) and sequentially increased. Firstly, developmental theory [69] and Stodden and colleagues’ conceptual framework [35] indicated that age plays a significant role in perceived competence. Younger children often lack the cognitive abilities to differentiate AEI; as a result, they frequently overestimate their intensity levels [70,71]. After growing into their adult forms, kids acquire more cognitive skills and can better recognize how much physical exercise they are doing [36]. Hence, we gathered and reviewed many papers on RPE in OW and OB individuals of various ages. Subgroup analysis was conducted based on age groups (i.e., ≥18 and <18). The findings revealed no heterogeneity within the age group of ≥18, but a low degree of heterogeneity (I^2^ = 30%; *p* = 0.13) was seen in the age group of <18. In other words, the correlation coefficient analysis provided findings that were consistent with this finding, and the correlation value of age was the greatest. Adults in the OW/OB groups had greater validity for RPE than children, as evidenced by Goodway et al. [72] and Noordstar et al. [70], who found that children’s cognitive capacity is generally not strong and often overestimates exercise intensity [70,72]. Considering children’s cognitive ability level, we suggest that RPE should be used with caution in children. However, there was another finding in our study when we performed the sensitivity analysis. The research of Michael et al. [64] was highly influential, since there was no heterogeneity after removing it from the study sample. Following our analysis of this paper, we discovered that the experiment mode was a four-week training course. Throughout this course, the children’s perceptive ability significantly improved, resulting in a significant difference in scores. Thus, we recommend that, when children use RPE, they are trained in their perception in advance. Additionally, it should be emphasized that we discovered a positive correlation between age and the RPE scores, as the latter rose with the former. Hence, RPE scores should be greater in adults than in children, but this result is contrary to our hypothesis that young people would overestimate them. Because the scale used for ages > 18 in the included studies was that of Borg (6–20), but the scales used for ages ≤ 18 were those of Borg (0–10) and OMNI, the Borg (6–20) values were greater than the values of Borg (0–10) and OMNI, which explains the contradictory findings.

The correlation analysis shows that BMI was correlated with RPE scores, which was consistent with the meta-analysis results. Nonetheless, OW and OB participants had higher RPE scores than those of normal weight, resulting in an overestimation of the intensity of their exercise. However, the RPE scores showed no significant differences between normal weight and overweight/obese individuals. The validity and reliability of RPE were verified in OW and OB people. The BMI of a person who is either normal or overweight varies with age and sex, increasing with age [73]. In order to determine whether BMI alters the link between AEI and RPE, it would be excellent to evaluate populations of the same age and sex with different BMI values. In this review, only the factor of age was considered, so the influence of BMI on the same gender was not considered, because we could not locate any all-male studies throughout the inclusion process.

The NW and OW/OB groups showed inconsistent correlations in test time and control temperature for the exercise protocol. Test time was associated with RPE scores in the NW group. The interval between RPE tests and exercise can cause recall bias [74], and the heart rate of NW individuals recovers faster than that of obese people [75]. Therefore, RPE scores are more distinctly affected by the time test in the NW group [75]. Control temperature was found to be associated with RPE scores in the OW/OB group. Obese individuals are more sensitive to temperature perception than NW individuals due to their very dense adipose tissue under the skin, which impedes heat dissipation during the summer. Hot weather and low intensity activity may cause obese individuals to feel out of breath and sweaty. They often need to use their lungs to assist in dispersing body energy [76,77,78]. This is why we have concluded that controlling temperature is required to maintain the accuracy of the experiment conclusion. Another prediction was that correlations would be greater in various types of exercise and protocols [34]. Concerning exercise selection, we discovered that the assumption that exercise choice is related to perceived scores was incorrect in normal and obese individuals. However, it is still worth emphasizing that though the experiment movement mode is predetermined, when the RPE is used to predict the research subject activity intensity (e.g., housework, transportation, and work), it may result in different conclusions because the patterns are not fixed. Even though exercise type was not found to be associated with RPE in this study, the effect of the type of exercise should not be ignored when using RPE. Additionally, we found that RPE scores were not related to the intensity selection of the exercise protocol. However, low exercise intensity was reported in eight reviewed studies (n = 617; 83.3%). It was not possible to conduct longitudinal analysis with moderate and vigorous intensity. Hence, one conclusion was drawn from this analysis: at low intensity, the AEI of OW/OB individuals matches RPE scores. Further research is needed at moderate and high intensities.

### 4.2. Summary of Risk of Bias from Included Studies

While the current systematic review and meta-analysis findings are noteworthy, it is recommended that their conclusions be interpreted with caution due to the risk of bias reported in eleven of the included studies. Except for that of Hassan et al. [57], the research was found to be dubious regarding at least one risk of bias criterion. Bias was found to mainly come from the inconsistency of the design requirements for reliability and measurement errors, as well as the sample size used in PROM development. For instance, reliability and measurement errors indicated that it was unclear whether test conditions were comparable; only two publications questioned diet, and four studies established a standard temperature. It has been shown that the use of certain chemical substances (e.g., coffee, citrulline, and alcohol) may cause perception to be altered [79,80]. In the preceding section, the impact of temperature was addressed. It is thus recommended to either rely on prior research to select reliable and valid design requirements for the intended BMI group (and explicitly refer to this prior research) or conduct pilot studies to assess the validity and reliability of the design requirements being used before setting up larger-scale studies. A significant risk of bias originated from the sample size employed in four of the included studies. Just five studies (45.5%) had an acceptable sample size. This is a grave problem and illustrates the general weakness of research in this field. These findings indicate the need to increase awareness among journal editors, reviewers, and authors about the importance of providing comprehensive information on each of these quality criteria to enable more rigorous meta-analyses that are less prone to bias in the future.

### 4.3. Summary of Heterogeneity from Included Studies

We have outlined the causes of heterogeneity based on the results of the bias assessment and hypothesis verification procedures. Firstly, heterogeneity mainly appeared in the children’s group. In the low age category (younger than 18), the mean age was 8 years. According to cognitive theory, the cognitive capacity of this cohort has not yet been developed to a high degree [81,82]. Second, according to the statistical concept, a sample of fewer than 30 people is deemed a tiny sample [83,84], which results in a significant error due to sample size [85,86]. Thirdly, acquired training was found to have an impact on children’s motor perception [87,88]. The fourth finding was that the mean BMI values in the included articles varied, including those that were OW and OB, and the RPE scores increased with BMI. Finally, throughout the experimental design procedure, the diet and temperature settings were inconsistent.

### 4.4. Practical Implications and Directions for Future Research

Overall, while the objective of a systematic review and/or meta-analysis is to present a clearer picture of research in a particular field of study, this review highlights the lack of clarity in some aspects of AEI and RPE in people who are overweight and obese. Many possible moderating variables contribute to the intrinsic complexity of the connection between AEI and RPE. Clearly, a systematic approach to research in this field is necessary to uncover the underlying elements inherent in how individuals perceive their own exercise intensity. Among various self-concept aspects, RPE has been found to be the most effective predictor of exercise intensity among normal weight individuals [30]. As a result, addressing the validity, reliability, and accuracy of RPE evaluation and its connection to AEI is critical in the OW and OB groups, because the test technique is simple, not time-consuming, and can effectively conserve human and material resources. Furthermore, RPE assessment of exercise intensity is critical in creating exercise prescriptions, directing scientific exercise, training, cardiac rehabilitation, mass fitness, and competitive sports [89,90]. For example, while engaging in physical exercise, usually healthy individuals may use the RPE table to regulate their exercise intensity. RPE may be used to conduct a post-exercise or rehabilitation training evaluation for OW and OB patients or other kinds of patients, serving as a guideline for establishing an acceptable and efficient exercise prescription [91,92]. Some significant research avenues may be pursued in the future.

Future researchers interested in this topic may wish to investigate alternative strategies for increasing the reliability of longitudinal comparisons on the moderating role of age and gender in the relationship between AEI and RPE. Additionally, in order to accurately analyze the influence of moderate and vigorous intensity exercise, research with different exercise intensities in the same population is required. Finally, we suggest seeking to determine whether there are additional confounding variables that could affect the relationship between AEI and RPE, such as the method by which AEI is assessed in real-world settings rather than in the laboratory (i.e., process-oriented measures) and cross-cultural effects (e.g., ethnicity and culture).

### 4.5. Strengths and Limitations

This systematic review and meta-analysis were comprehensive. We found the reasons for the heterogeneity of the controversial research results and addressed multiple potential modifiers of the link between AEI and RPE in people with OW or OB. Additionally, we increased the reliability and validity of RPE in OW and OB populations. The authors performed searches without specifying a year or an age range. Though this article was not written in any of the author’s original languages, in order to ensure the accuracy and logic of the language used in the study, we sent it to colleagues whose native tongues corresponded to the written language of the study (as noted in the Acknowledgements section) and applied for the language improvement service of a professional agency. Therefore, we are sure that the search provides an accurate picture of the literature. Multiple variables were found to potentially affect the connection between AEI and RPE using a thorough investigation that considered the impact of possible confounding factors. The limited number of studies in the collegiate and senior age groups is a limitation of the study. We cannot establish gender impacts on OW and OB individuals due to an all-male research paucity. In the literature, only a small number of studies utilized moderate and vigorous intensity exercise. Thus, we could not conclude regarding the impact of moderate and vigorous intensity exercise on RPE, and there was an absence of relevant research after February 2019. Our focus on English likely resulted in the marginalization of relevant and significant literature in other languages. Finally, although the meta-analyses included various important moderators, it was not feasible to include every conceivable moderator in the analysis.

## 5. Conclusions

According to the findings of this in-depth systematic review and meta-analysis, an association between AEI and RPE in OW and OB people was found in RPE scores, which were higher than in the NW group. Still, there was no statistically significant difference between the two groups based on the currently implemented methodologies of AEI and RPE. The associations of these constructions are usually distinguished by age, temperature (only in the OW group), BMI, and test time (only in the NW group). As a result, we conclude that RPE is appropriate for the following OW and OB people: (1) those who are older than 21.5 (the lowest age in the group of 18) years old, younger than 58.6 (the highest age in the group of 18) years old, and without any other diseases, and (2) those who engage in low intensity exercise while maintaining a standard indoor temperature.

## Figures and Tables

**Table 1:** Overview of selected reviews in alphabetical order (n = 11).

Reference	Country	Sample Size (M^1^/F^2^)	Mean Age (Years)	Mean BMI	Exercise Modality	Exercise Protocol	Physiological Criterion	PE ^9^ Scale
Connie et al. 2015 [55]	USA	52 (34/18)	NW ^3^: 8.6 SD: 0.9 OB: 8.7 SD: 0.7	NW: 16 SD: 1.2 OB: 23 SD: 1.3	45 min school PA	Vigorous Continuous	HR	Borg scale (6–20) Test time: end of exercise
Ekkekakis et al. 2005 [56]	USA	25 (25/0)	NW: 43.67 SD: 4.24 OW: 43 SD: 5.4	NW: 22.34 SD: 1.82 OW: 31.06 SD: 4.91	20 min treadmill exercise	Low Intermittent	VO_2peak_ and HR	Borg scale (6–20) Test time: each 5 min
Hassan et al. 2013 [57]	Brazil	66 (66/0)	NW: 30.8 SD: 9.3 OB: 33.5 SD: 8.5 OW: 34.8 SD: 8.6	NW: 22 SD: 1.6 OB: 34.9. SD: 4.1 OW: 26.4 SD: 1.3	Maximal graded treadmill	Low Continuous Standard temperature	HR_max_, VE_max_, VO_2VT_, %VO_2_ ^26^, VT_max_, %HR_max_ ^25^, VT, HRVT, VO_2max_, and RER_max_	Borg scale (6–20) Test time: the last 15 s
Hassan et al. 2018 [58]	Brazil	66 (66/0)	NW: 30.8 SD: 9.3 OB: 33.5 SD: 8.5 OW: 34.8 SD: 8.6	NW: 22 SD: 1.6 OB: 34.9. SD: 4.1 OW: 26.4 SD: 1.3	GXT	Low Intermittent	HR andVO_2_	Borg scale (6–20) Test time: the last 15 s
Jeremy et al. 2009 [59]	USA	43 (43/0)	OB: 50.5 SD: 8.1	OB: 39.6 SD: 6.1	GXT	Moderate Continuous Standard temperature	VO_2max_ ^27^	Borg scale (6–20) Test time: the last 15 s
Karen et al. 2006 [60]	Australia	22 (19/3)	OB: 45.7 SD: 9.8	OB: 33.6 SD: 2.6	Submaximal cycle test	Low Intermittent aerobic, and power index Standard temperature	VO_2_	Borg scale (6–20) Test time: recorded 55 s
Hulens et al. 2003 [61]	Belgium	300 (300/0)	NW ^3^: 39 SD ^7^: 11.8 OB ^5^: 39.5 SD: 11.4 OB morbidity: 38.9 SD: 12.4	NW: 22.1 SD: 2.1 OB: 32.3 SD: 1.9 OB morbidity: 40.7 SD: 4.4	GXT ^6^	Low Continuous	VO_2_peak ^17^ and HR ^10^	Borg scale (6–20) Test time: the last 15 s
Marinov et al. 2002 [62]	Bulgaria	60 (30/30)	NW: 11.0 SD: 3.1 OB: 10.9 SD: 3.1	NW: 18.8 SD: 2.7 OB: 27.4 SD: 4.5	Cardiopulmonary exercise test	Moderate Continuous	VT^14^, BF ^15^, VE ^12^, VE⁄ MVV ^16^, VO_2_peak, VO_2_ ^11^, VO_2_⁄FFM ^18^, VO_2_⁄BSA ^19^, VO_2_⁄HR ^20^, HR, RER ^21^, AT ^24^, VE/VO_2_ ^23^, VE ⁄ VCO_2_ ^22^, and VCO2/VO2 ^13^	Borg scale (0–10) Test time: end of exercise
Matthew et al. 2019 [63]	USA	21 (12/9)	NW: 9.4 SD: 0.5 OW ^4^: 10.2 SD: 0.4 NW: 10.3 SD: 0.6 OW: 10.5 SD: 0.7	NW: 16.3 SD: 1.7 OW: 23.1 SD: 2.2 NW: 16 SD: 2 OW: 23.5 SD: 1.9	Self-selected: 20 min submaximal cycling	Low Continuous	HR VO_2_/VO_2_peak 70%peak HR	OMNI scale Test time: each 5 min
Michael et al. 2008 [64]	Australia	61 (28/33)	OB: 9.7 SD: 1.4	OB: 25.6 SD: 3.4	Resistance exercise	Low Intermittent	NA ^8^	OMNI scale Test time: end of exercise
Mostafa et al. 2019 [65]	New Zealand	25 (13/12)	NW: 12.23 SD: 1.23 OW: 12.1 SD: 1.22	NW: 17.35 SD: 2.64 OW: 28.7 SD: 7.05	Butt kick Stationary running Frontal kick	Low Intermittent Standard temperature	HR	Borg scale (6–20) Test time: end of exercise

Note: ^1^ M: Male. ^2^ F: Female. ^3^ NW: normal weight. ^4^ OW: overweight. ^5^ OB: obese. ^6^ GXT: grade exercise test. ^7^ SD: standard deviation. ^8^ NA: not available. ^9^ PE: perceived exertion. ^10^ HR: heart rate. ^11^ VO_2_: oxygen uptake. ^12^ VE: minute ventilation. ^13^ VCO_2_/VO_2_: respiratory ratio. ^14^ VT: tidal volume. ^15^ BF: breathing frequency. ^16^ VE/MVV: minute ventilation/maximal voluntary ventilation ratio; ^17^ VO_2_peak: peak oxygen uptake. ^18^ FFM: fat-free mass. ^19^ BSA: body surface area. ^20^ VO_2_/HR: oxygen pulse. ^21^ RER: respiratory exchange ratio. ^22^ VE/VO_2_: ventilatory equivalent for oxygen. ^23^ VE/VCO_2_: ventilatory equivalent for carbon dioxide. ^24^ AT: anaerobic threshold. ^25^ %HR_max_: percentage of maximal heart rate. ^26^ %VO_2_: percentage of maximal oxygen uptake.^27^ VO_2max_: maximal oxygen uptake.

## Data Availability

Not applicable.
